# Multiple subregions within the caveolin-1 scaffolding domain inhibit fibrosis, microvascular leakage, and monocyte migration

**DOI:** 10.1371/journal.pone.0264413

**Published:** 2022-02-25

**Authors:** Charles F. Reese, Panneerselvam Chinnakkannu, Elena Tourkina, Stanley Hoffman, Dhandapani Kuppuswamy

**Affiliations:** 1 Division of Rheumatology, Department of Medicine, Medical University of South Carolina, Charleston, South Carolina, Unites States of America; 2 Division of Cardiology, Department of Medicine, Medical University of South Carolina, Charleston, South Carolina, Unites States of America; University of Tennessee Health Science Center College of Medicine Memphis, UNITED STATES

## Abstract

The caveolin-1 scaffolding domain (CSD, amino acids 82–101 of caveolin-1) has been shown to suppress bleomycin-induced lung and skin fibrosis and angiotensin II (AngII)-induced myocardial fibrosis. To identify active subregions within CSD, we split its sequence into three slightly overlapping 8-amino acid subregions (82–89, 88–95, and 94–101). Interestingly, all three peptides showed activity. In bleomycin-treated mice, all three subregions suppressed the pathological effects on lung and skin tissue morphology. In addition, while bone marrow monocytes isolated from bleomycin-treated mice showed greatly enhanced migration in vitro toward CXCL12, treatment in vivo with CSD and its subregions almost completely suppressed this enhanced migration. In AngII-induced heart failure, both 82–89 and 88–95 significantly suppressed fibrosis (both Col I and HSP47 levels), microvascular leakage, and heart weight/ body weight ratio (HW/BW) while improving ventricular function. In contrast, while 94–101 suppressed the increase in Col I, it did not improve the other parameters. The idea that all three subregions can be active depending on the assay was further supported by experiments studying the in vitro migration of human monocytes in which all three subregions were extremely active. These studies are very novel in that it has been suggested that there is only one active region within CSD that is centered on amino acids 90–92. In contrast, we demonstrate here the presence of other active regions within CSD.

## Introduction

Caveolin-1 is a structural cytoplasmic membrane protein involved in the formation/function of caveolae [1]. Besides its role in endocytosis and receptor trafficking, caveolin-1 is a master regulatory protein that inhibits a wide range of kinases and also eNOS and NADPH oxidase by binding to these proteins via the caveolin-1 scaffolding domain (CSD, amino acids 82–101) [2–9]. CSD treatment is beneficial in mouse model systems (e.g. lung fibrosis, heart failure) in that it suppresses fibrosis, microvascular leakage, and inflammation. These effects are mediated by CSD’s ability to act as a surrogate for caveolin-1, thereby reversing the effects of low caveolin-1 associated with fibrosis in vitro and in vivo in fibroblasts, monocytes, and mesenchymal stem cells [8, 10–17]. In contrast, in endothelial cells CSD may have beneficial effects by acting as a competitor of the function of caveolin-1. This dual function of CSD has been suggested previously by many authors [18–20].

In previous studies, we have shown the beneficial effects of CSD treatment in multiple mouse models: heart failure (HF) induced by either transverse aortic constriction or angiotensin II (AngII) infusion [21, 22]; kidney fibrosis induced by AngII [21]; bleomycin-induced lung and skin fibrosis [12, 13, 15, 16, 23]; and aging-associated fibrosis of the heart, kidney, and brain [24]. These observations make CSD a rational candidate for development as a treatment for the many human diseases involving organ fibrosis and/or microvascular leakage (e.g. HF [14], renal failure [25], interstitial lung diseases (e.g. scleroderma [systemic sclerosis, SSc] and idiopathic pulmonary fibrosis) [26–28], Alzheimers’ disease and other neurodegenerative diseases [29–32], aging-associated diseases [33], cancer [19, 34], asthma [35], complications of diabetes [36], sepsis [37], and acute respiratory distress syndrome (ARDS) which is the major cause of death in Covid-19 infection [38–41].

To better understand the structure-activity relationship of CSD and to promote its development as a drug, here we have evaluated the activity of three independent subregions (amino acids 82–89, 88–95, 94–101) of caveolin-1. In general, shorter peptide drug candidates such as these are advantageous because they are easier to synthesize and should exhibit increased tissue penetration and decreased immunogenicity and other side effects [42, 43]. While some previous literature predicted that only 88–95 would have beneficial effects [44–46], we present extensive evidence here that all three subregions can be active.

## Methods

### Animals

Mice were maintained at constant room temperature (20–22°C) with free access to water and food under a 12:12 h light: dark cycle. All animal experiments were performed under a protocol approved by the Medical University of South Carolina (MUSC) Institutional Animal Care and Use Committee (IACUC).

### Bleomycin treatment

Ten-week old, C57/BL6 mice (Charles River Laboratories, Boston, MA) were maintained under pathogen-free conditions. Briefly, osmotic minipumps (ALZET 1007D; DURECT Corporation, Cupertino, CA) containing either 100 μl saline vehicle or bleomycin (100 U/kg) designed to deliver their contents at 0.5 μl/h for 7 days were implanted under isofluorane anesthesia under the loose skin on the back of the mice slightly caudal to the scapulae as we have described [47]. Pumps containing bleomycin or vehicle were removed on day 8 and replaced with pumps containing the indicated peptides (n = 6 per group). After 14 more days, mice were sacrificed by isoflurane overdose, systemically perfused with PBS via the left ventricle, and lung and skin tissue harvested. Skin tissue was harvested adjacent to the main body of the pump.

For histology, lung lobes were perfused with buffered zinc formalin fixative (Z-Fix; Anatech, Battle Creek, MI). Lung lobes and skin strips were further fixed overnight in Z-Fix, then embedded in paraffin. Sections (4 μm) were stained with hematoxylin and eosin (H&E) or Masson’s trichrome or immunohistochemically.

### Monocyte migration

Bone marrow (BM) monocytes were isolated for use in migration experiments as we described [21]. In these experiments, both activation (due to bleomycin treatment) and treatment with CSD and subregions occurs in vivo. Healthy human monocytes were isolated and activated with TGFβ (45 min, 10 ng/ml in RPMI 1640/1% BSA) as we described [15].

Migration experiments with both mouse and human monocytes were performed as we have described [15]. Briefly, CXCL12 (100 ng/ml in RPMI 1640/1% BSA) or unsupplemented RPMI 1640/1% BSA was placed into the lower wells of Neuro Probe Multiwell Chemotaxis Chambers (Neuro Probe, Gaithersburg, MD) fitted with 5-μm pore size polycarbonate filters. 25 μl of cell suspension (5 × 10^5^ cells/ml) was placed in the upper wells. Peptides at the indicated concentrations were added to the cell suspension prior to placement. After incubation (2.5 h, 37° C, 5% CO_2_), filters were removed, fixed, and stained with 4′,6-diamidino-2-phenylindole (DAPI) (Invitrogen, Carlsbad, CA). Cells on the underside of the membrane were photographed and counted in six high power fields per condition.

### AngII treatment

Wild-type C57BL/6J male mice (3 months) were purchased from Jackson Laboratory (Bar Harbor, Maine). Mini-osmotic pumps (ALZET 1002; DURECT Corporation, Cupertino, CA) were implanted as described above. The pumps contained either 100 μl saline or AngII (2.1 mg/kg/day) and delivered their contents at 0.25 μl/h for 2 wk. After two weeks, heart function was evaluated by echocardiography. Mouse sacrifice and sequential preparation of RIPA-soluble and RIPA-insoluble fractions for Western blotting were performed as previously described [22, 48]. As in previous studies, HSP47 and IgG Heavy Chain Western blots were performed using the soluble fraction and Col I Western blots were performed using the insoluble fraction. This sequential extraction provides better resolution by SDS-PAGE and the enrichment of these proteins in the fractions in which they are most abundant. Measurements of heart weight (in mg) and body weight (in g) were used to calculate the HW/BW ratio as previously described [22, 48].

### Echocardiography

Echocardiography was performed using a Vevo2100 imaging system (VisualSonics, Toronto) as we described [22, 48]. Briefly, isoflurane anesthesia was used and heart rate was maintained at 400–500 bpm. Two-dimensional B- and M-mode analyses were performed using the 22–55 MHz linear transducer probe and VisualSonics 1.2.0 software. M-mode images of the parasternal short-axis view at papillary level was used to calculate ejection fraction (EF) and fractional shortening (FS). B-mode images of the parasternal long-axis were used to calculate stroke volume (SV) and cardiac output (CO). Tissue Doppler was used in the short-axis view at the papillary muscle level to obtain velocity of posterior left ventricular (LV) wall motion. 5 to 10 cycles were recorded to calculate isovolumic relaxation time (IVRT).

### Peptides

The sequence of peptides purchased from Elim Biopharmaceuticals (Hayward, CA) are shown in Table 1. Peptides were dissolved in DMSO. In the bleomycin experiment, peptides in DMSO were diluted 10-fold with saline so that osmotic minipumps loaded with 100 μl delivered 8 μmol/kg over two weeks. In the AngII experiment, peptides in DMSO were diluted 100-fold with saline so that daily i.p. injections of 100 μl delivered 0.8 μmol/kg per day.

**Table 1:** Sequences of CSD, subregions, and scrambled CSD.

CSD(82–101) DGIWKASFTTFTVTKYWFYR -NH2 82–89 DGIWKASF -NH2 88–95 SFTTFTVT -NH2 94–101 VTKYWFYR -NH2 Scrambled CSD ***W***GI***D***KA***F***FTT***S***TVT***YK***WF***RY*** -NH2

Amino acids in altered positions in Scrambled CSD are in ***Bold Italics***.

### Statistical analyses

Values are presented as mean ± SEM. Differences were analyzed between groups using one-way analysis of variance (ANOVA) followed by a *post hoc* Tukey’s multiple comparison to determine statistical significance.

## Results

We previously demonstrated that CSD (aa 82–101 of caveolin-1) has a wide range of beneficial effects in two distinct mouse heart failure models (TAC and AngII-induced disease) and in bleomycin-induced lung [16] and skin fibrosis [12]. In order to evaluate the Structure-Activity Relationship for CSD, we have divided it into three, slightly overlapping peptides of equal length (82–89, 88–95, and 94–101) and tested them in multiple systems in vivo (mouse lung and skin fibrosis, heart fibrosis) and in vitro (human monocyte migration). Whereas some previous studies concluded that only 88–95 should be active [46], we find significant activity in all three subregions.

### Suppression of bleomycin-induced lung fibrosis by CSD subregions

We found that delivering bleomycin to mice via subcutaneously implanted osmotic minipumps results in a disease very similar to human SSc [47]. These similarities include: both lung and dermal fibrosis are observed, the lung fibrosis is accompanied by relatively little inflammation as compared to when bleomycin is delivered intratracheally, and dermal fibrosis is accompanied by a loss of transdermal fat.

**Fig 1:**
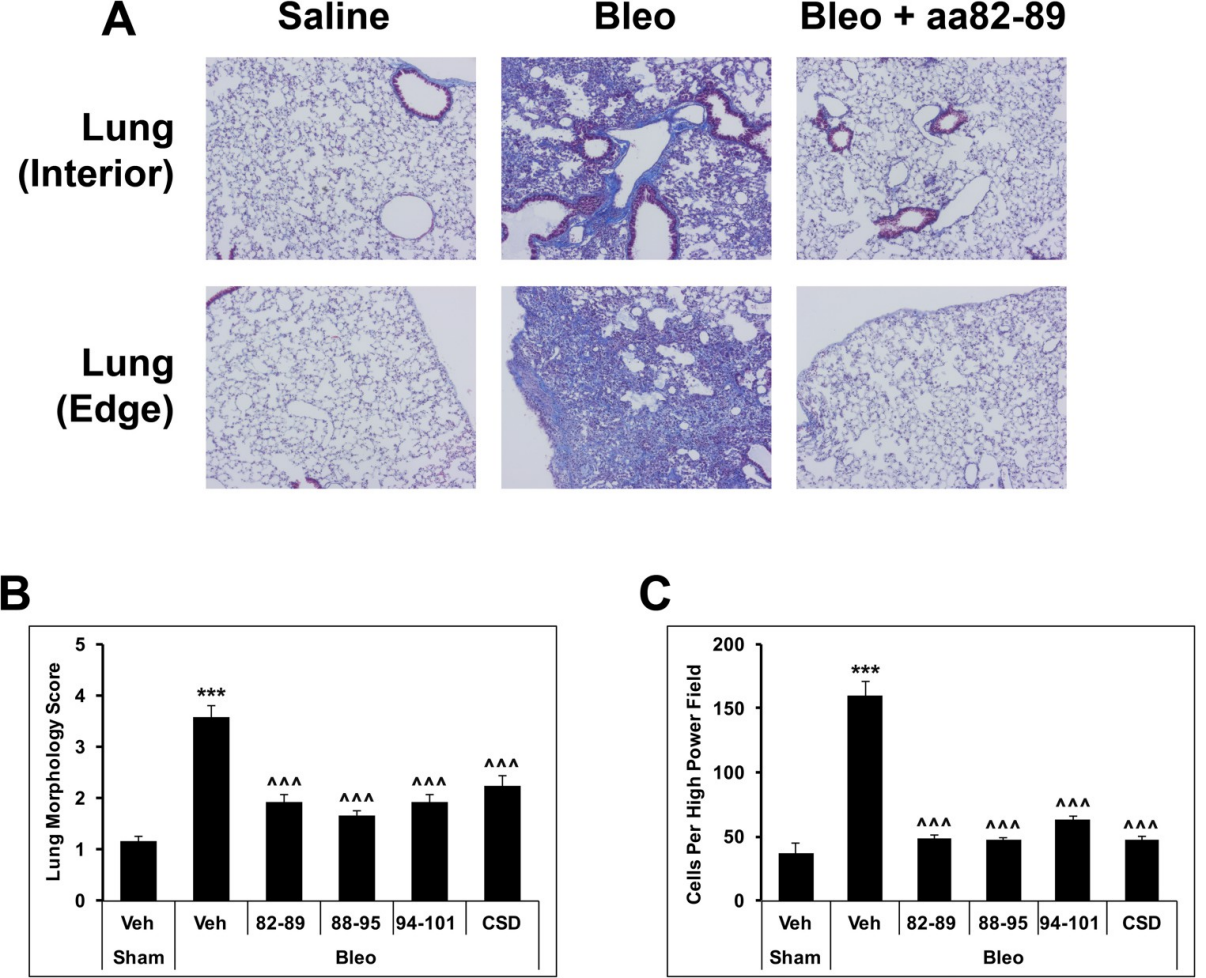
Inhibition of bleomycin-induced lung fibrosis and monocyte migration by subregions of CSD. A disease state similar to human SSc was induced by systemic treatment of mice with bleomycin [47]. Following removal of the bleomycin-containing pumps on day 8, treatment was initiated using pumps implanted subcutaneously loaded with 8 μmol/kg of the indicated peptides that release their contents for 2 weeks. **(A)** Masson’s Trichrome-stained lung tissue sections demonstrate the massive fibrosis caused by bleomycin and its reversal by 82–89 both in the edge and interior of a lung lobe. **(B)** Tissue Morphology in Masson’s Trichrome-stained tissue sections was quantified as described [16, 49]. (n = 6 per group) **(C)** Monocyte migration in vitro was quantified as described [21] using BM monocytes isolated from mice treated in vivo both with bleomycin and with peptides as indicated. (n = 4 per group) *** p<0.001 vs Sham control; ^^^ p<0.001 vs Bleo control.

To determine the beneficial effects of CSD subregions on bleomycin-induced lung fibrosis, when pumps delivering bleomycin were removed seven days after implantation, they were replaced with pumps delivering CSD or the subregions for the next two weeks. This is a therapeutic protocol because the potentially beneficial treatments are begun only after disease has been induced. Images of tissue morphology are shown in Fig 1A and quantified in Fig 1B. These data demonstrate that full-length CSD (82–101) as well as subregions 82–89, 88–95, and 94–101 all are extremely beneficial.

To determine whether this effect might involve the inhibition of the migration of BM-derived monocytes into the lung, BM monocytes were isolated from these six groups of mice and their ability to migrate toward the chemokine CXCL12 in vitro was determined. The migration of BM monocytes from bleomycin-treated mice was enhanced four-fold compared to control, saline-treated mice. This enhancement of migration was almost completely reversed in mice treated in vivo with CSD, 82–89, 88–95, or 94–101 (Fig 1C).

**Fig 2:**
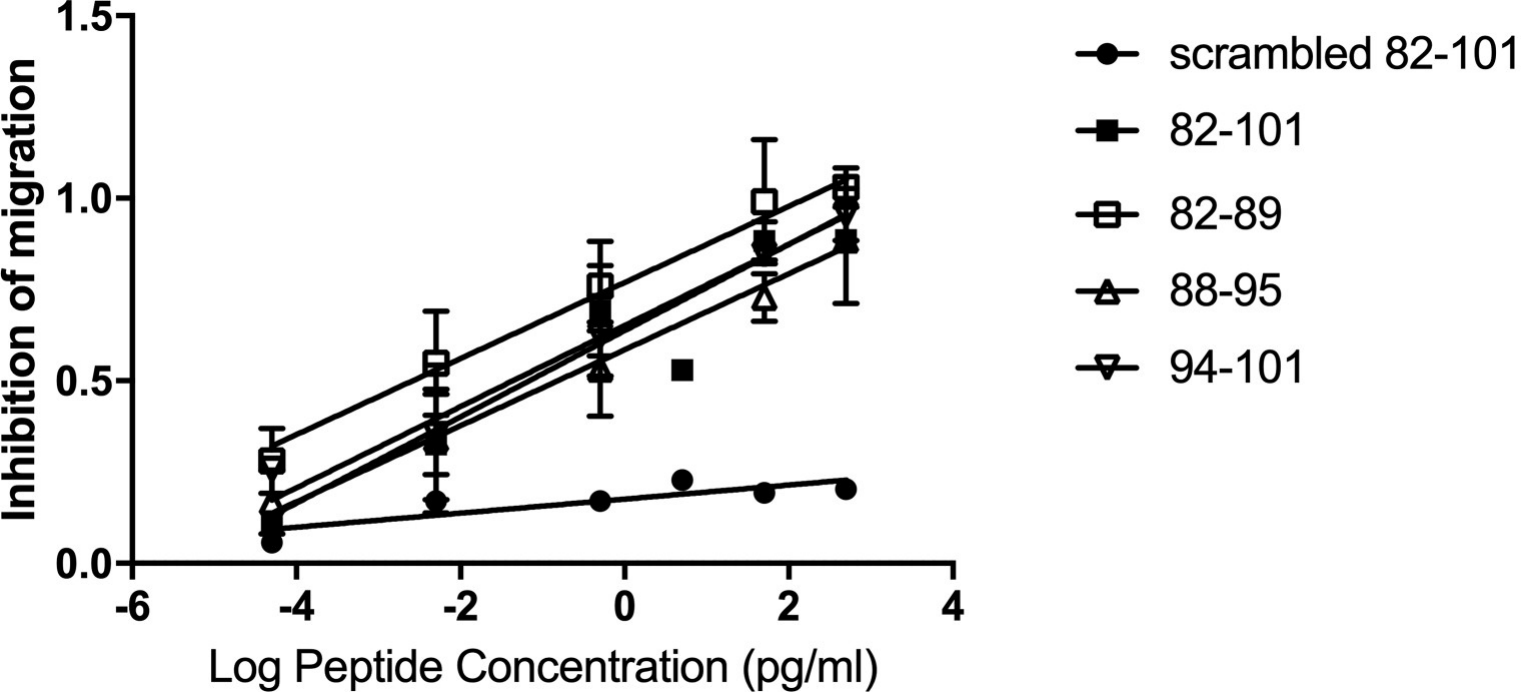
Dose-dependent effect of CSD subregions on human monocyte migration. Healthy human monocytes were treated with the indicated versions and concentrations of CSD. 100 ng/ml SDF-1 was the chemoattractant. Migrating cells were counted in 6 high power fields per filter. Each experiment included control (unstimulated) monocytes and the same monocytes treated with TGFβ, which enhanced their migration ~3-fold. Inhibition of migration is presented on a linear scale of 0.0 (no effect) to 1.0 (reduction to level observed in absence of TGFβ). Results are summarized from 11 independent experiments.

To validate this effect of CSD subregions on monocyte migration using human cells, peripheral monocytes were isolated from healthy donors and activated using TGFβ. CSD and all three subregions exhibit dose-dependent inhibition of TGFβ-induced migration even at extremely low concentrations while scrambled CSD (Table 1) was inactive (Fig 2). Again, 82–89 was the most active while CSD, 88–95, and 94–101 were similar in activity to each other.

### Suppression of bleomycin-induced dermal fibrosis and restoration of the intradermal fat layer by CSD subregions

**Fig 3:**
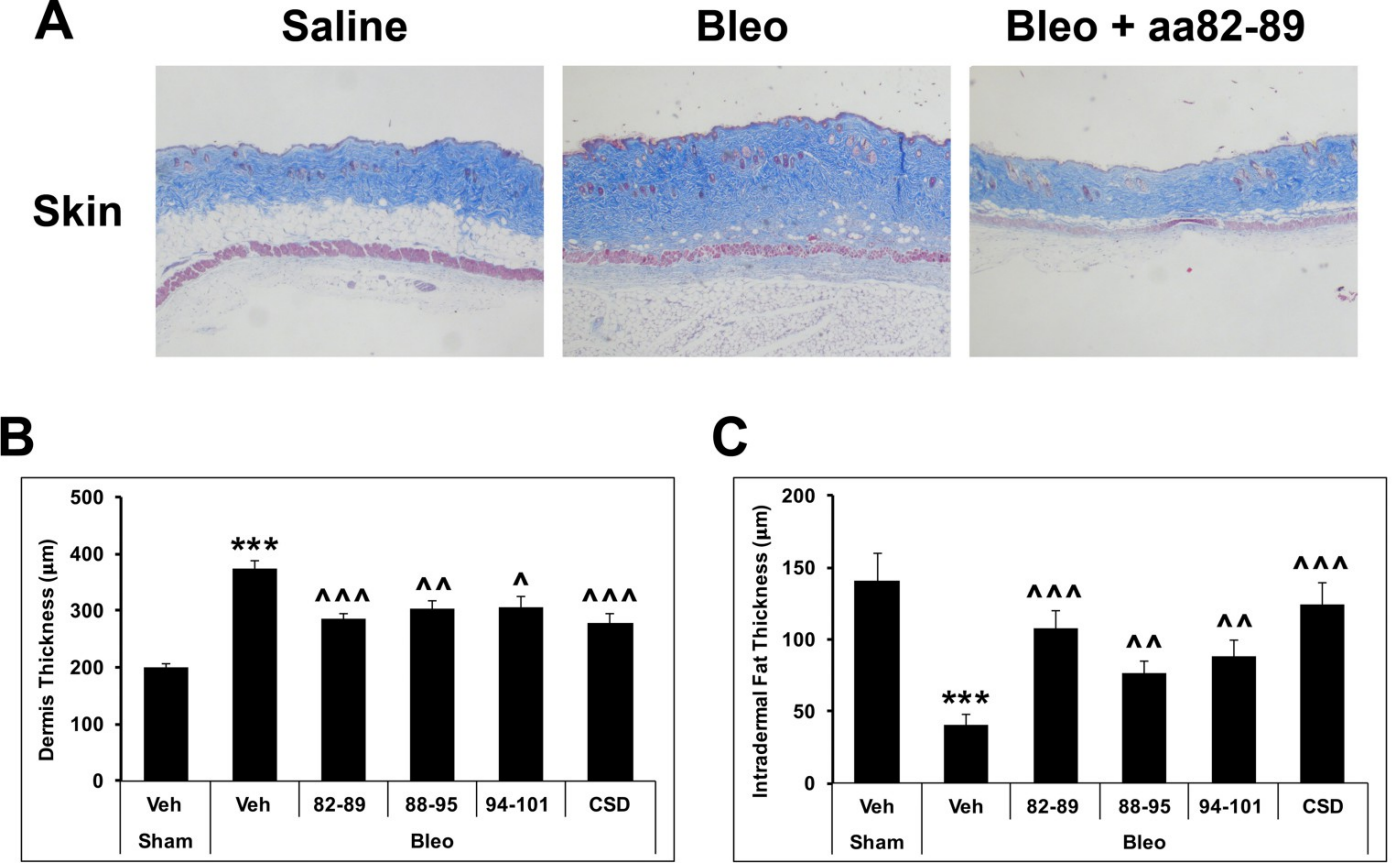
Inhibition of bleomycin-induced dermal fibrosis and thinning of the transdermal fat layer (lipoatrophy) by subregions of CSD. The same mice used in Fig 1 were used here. After sacrifice, the thickness of the dermis and intradermal fat layer in tissue sections were measured (six mice per condition). **(A)** Masson’s Trichrome-stained skin tissue sections demonstrate the dermal fibrosis and lipoatrophy caused by bleomycin and its reversal by 82–89. **(B)** Quantification of dermal fibrosis. **(C)** Quantification of lipoatrophy. ***p<0.001 vs Saline control; ^^^p<0.001, ^^p<0.01, ^p<0.05 vs Bleo control.

Bleomycin causes a remarkable thickening of the dermis and thinning of the intradermal fat layer (lipoatrophy) in mice [12] just as occurs in SSc patients [50]. While all versions of CSD tested were effective in suppressing dermal thickening and lipoatrophy, CSD and 82–89 were the most effective (Fig 3).

### AngII model

**Fig 4:**
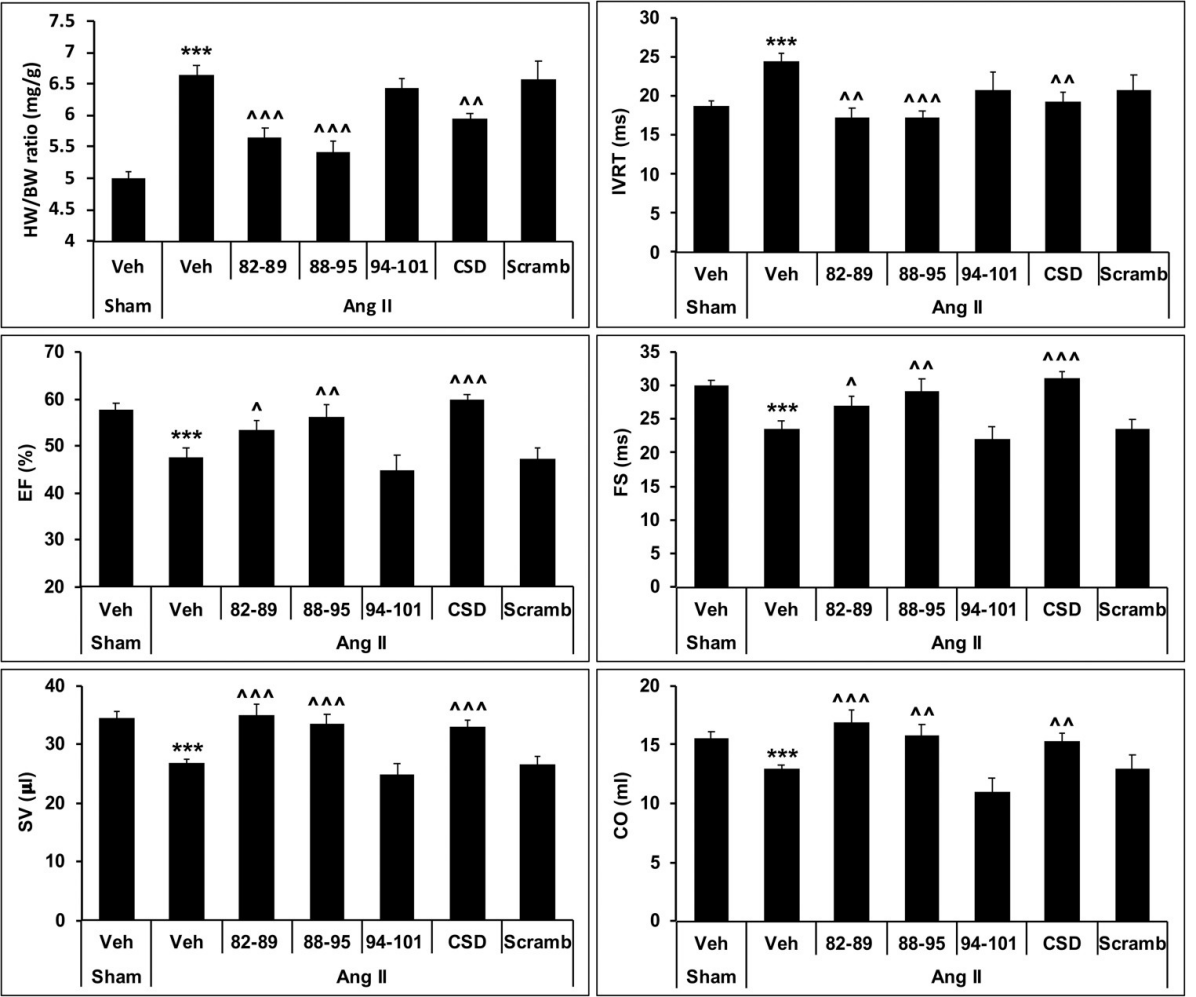
CSD and two subregions suppress Ang II-induced cardiac hypertrophy and pathological changes in ventricular function. Mice were infused for 2 weeks with Ang II (2.1 mg/kg/day) to induce HF or with vehicle. Concomitantly, CSD, scrambled CSD, or subregions were injected daily (i.p., 0.8 μmol/kg). Cardiac hypertrophy was evaluated in terms of the HW/BW ratio. Cardiac function was evaluated by echocardiography for IVRT, EF, FS, SV, and CO. Significant changes are shown as ***p<0.001 vs Sham control. Suppression of AngII-induced changes is shown as ^p<0.05, ^^p<0.01, and ^^^p<0.001 vs AngII control. Additionally, representative images of echocardiography data are provided in the Supplementary figures.

We have previously demonstrated that AngII-treated mice exhibit heart failure in terms of cardiac hypertrophy, fibrosis, microvascular leakage, and ventricular function and that all of these effects are suppressed by CSD [21]. When we evaluated 82–89, 88–95, and 94–101 in the AngII model, we found that 82–89 and 88–95 were effective in suppressing the pathological effects of AngII on cardiac hypertrophy (HW /BW ratio), IVRT, EF, FS, CO, and SV; while 94–101 and scrambled CSD were not effective (Fig 4).

**Fig 5:**
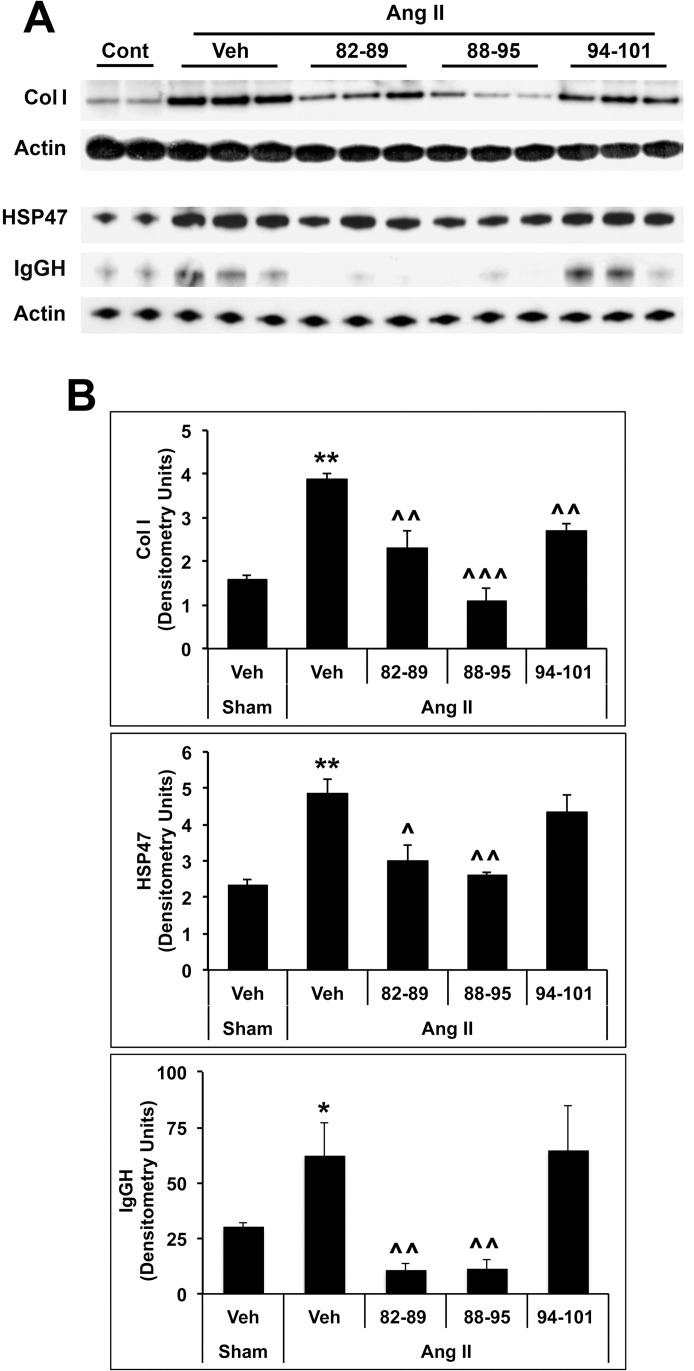
Inhibition of AngII-Induced fibrosis and microvascular leakage by CSD subregions. **(A)** HF induction and peptide treatment were as in Fig 4. Fibrosis was evaluated in LV tissue extracts by Western blot of Col I levels in the RIPA-insoluble fraction and HSP47 levels in the RIPA-soluble fraction. Microvascular leakage was evaluated in terms of IgG Heavy chain levels in the RIPA-soluble fraction. Actin in each fraction served as the loading control. n = 3 in this experiment. Similar results were obtained in a second experiment. **(B)** Quantification of Western blot results. Significant changes are shown as *p < 0.05 and **p<0.01 vs Sham control. Suppression of Ang II-induced changes by subregions are shown as ^p<0.05, ^^p< 0.01, and ^^^p<0.001 vs Ang II control.

We next evaluated fibrosis in terms of Col I and HSP47 levels and microvascular leakage in terms of IgG Heavy Chain levels (Fig 5). Again, CSD, 82–89, and 88–95 almost completely blocked the pathological changes in HSP47 and microvascular leakage while 94–101 had no effect. In contrast, all three subregions had a beneficial effect on Col I accumulation (Fig 5).

## Discussion

In previous studies, we have shown the beneficial effect of CSD treatment on heart fibrosis and function using the transverse aortic constriction (TAC) model [22], heart and kidney fibrosis using the AngII-infusion model [21], and lung and skin fibrosis induced by bleomycin treatment [12, 23]. Here, we are advancing these studies by performing translational research to develop subregions of CSD as treatments for heart, lung, and skin disease. CSD (amino acids 82–101) was recognized as early as 1996 [51] as the active domain of caveolin-1. Previous studies in both fibroblasts and endothelial cells have concluded that the active site within CSD centers on amino acids 90–92 [44, 46]. However, there are also clues in the literature for other active regions within CSD. For example, 86–101, but not 88–101, directly inhibited the activity of purified isoforms of Protein Kinase C [5] indicating the importance of the lysine and alanine at positions 86 and 87.

In accord with these previous studies, our peptide 88–95 is active in a variety of assays. However, we also find extensive evidence that our peptides 82–89 and 94–101 represent active subregions of CSD. In the bleomycin model, all three subregion peptides were effective in inhibiting lung fibrosis, skin fibrosis, and the migration in vitro of BM monocytes derived from these mice. Similarly, all three peptides inhibited the migration of human monocytes in vitro. In the AngII model for HF, both 82–89 and 88–95 were effective in suppressing the deleterious effects of AngII on HW/BW ratio, ventricular function (IVRT, EF, FS), fibrosis (Col I and HSP47 levels), and microvascular leakage although in some cases 88–95 is somewhat more active. While 94–101 did not show any beneficial effects in the AngII model in most assays, it did have a significant ability to inhibit Col I accumulation.

CSD peptides have been synthesized in different ways for use in different published studies. It was initially assumed both for studies in mice and in cell culture that attachment to the 16-amino acid insect Antennapedia Internalization Sequence was required for CSD to enter cells [19, 52]. However, it was later found that Antennapedia is not required for the biological effects of CSD both in vitro and in vivo [2, 20, 46].

Research groups have also differed in their capping of the C-terminus and N-terminus of CSD. For example, our peptides (Table 1) are capped at the C-terminus with an NH2 group while caps are not reported to be present on the peptides in a recent study whose results differed from ours [46]. In this study, 89–95 (called CSP7) was shown to inhibit fibrosis in vivo and in fibroblast cultures and to inhibit epithelial cell apoptosis in vivo, while 82–88 (called CSP3) and 96–101 (called CSP5) were inactive. Reasons that could be responsible individually or in combination to explain the difference between their results and ours include: 1) There are one or two amino acid differences between our peptides 82–89, 88–95, and 94–101 and their peptides 82–88, 89–95, and 96–101; 2) Our peptides are capped with a C-terminal NH2 group while their peptides are not reported to be capped; 3) Reasons “1” and/or “2” may lead to their peptides 82–88 and 96–101 not being able to enter cells; and 4) Although our 82–89 inhibits Col I accumulation in vivo, this may be an indirect effect due to its ability to inhibit microvascular leakage. In other words, the primary cellular target of 82–89 may be endothelial cells and it may have little direct effect on Col I expression by myofibroblasts. Thus, discrepancies between our study and other studies may depend on the presence or absence of modifications such as the Antennapedia sequence and/or C-terminal capping. In any case, it seems rather more likely that our inclusion of a C-terminal cap on 82–89 and 94–101 has revealed their beneficial effects as opposed to creating a false positive beneficial effect.

A key factor in understanding the function of CSD is the observation that CSD cannot form oligomers and cannot incorporate into the caveolin-1 oligomers that coat caveolae [53]. The CSD sequence within full-length caveolin-1 tethers a variety of kinases to plasma membrane caveolae or to the Golgi network and regulates their activities. However, free CSD may regulate kinases that are not membrane-bound or, alternatively, may cause kinases not to be tethered to the membrane via direct competition with CSD within full-length caveolin-1. The fact that the sites where CSD and its subregions can act are not limited to membranes may result in its activity at very low concentrations such as the inhibition of monocyte migration in vitro that we observed at sub-picogram levels of peptides (Fig 2).

Caveolin-1 and CSD regulate multiple molecular mechanisms relevant to microvascular leakage and fibrosis. The presence of IgG heavy chain in tissue extracts was used as a marker for microvascular leakage. Mechanisms underlying endothelial barrier function loss include eNOS/NO/ROS/ NADPH Oxidase signaling and tyrosine kinase signaling [54–56]. An increased ROS:NO ratio activates receptor (e.g. PDGFR, VEGFR) and non-receptor tyrosine kinases (e.g. Pyk2, c-Src, FAK) resulting in decreased barrier function and promoting microvascular leakage [6, 57–61]. Because caveolin-1 and CSD regulate eNOS, NADPH Oxidase, and non-receptor and receptor tyrosine kinases [3, 44, 51, 62], the ability of CSD and its subregions to suppress microvascular leakage may be mediated directly through its effects on tyrosine kinases or indirectly through its effects on NO:ROS signaling which in turn regulates tyrosine kinase activation.

We showed many years ago that caveolin-1 and CSD directly target fibrosis via pathways involving PKCα, PKCε, MEK, ERK, JNK, p38, and Akt that regulate Col I overexpression by myofibroblasts [8, 16]. Here we used Col I and the collagen chaperone HSP47 as markers for fibrosis. Besides the direct effects of CSD on fibrosis via these kinases, our more recent studies [21, 24] and the current study indicate that caveolin-1 and CSD also may indirectly regulate fibrosis via their effects on endothelial cells. When the permeability barrier function of endothelial cells is compromised, the resulting microvascular leakage promotes inflammation which may involve the recruitment of monocytes into injured tissue and their differentiation into fibrocytes/ myofibroblasts through mechanisms involving ERK, Smad 2/3, TGFβRI and RII, CXCR4, Lyn, and Src [63, 64]. In addition, fibrocytes secrete pro-fibrogenic factors that activate Col I expression by resident fibroblasts [65]. In summary, the ability of CSD to directly and indirectly regulate fibrosis involves multiple cellular and molecular targets. While it remains to be demonstrated, this complexity could further involve subregions of CSD differentially affecting various targets. Further, this differential effect could depend on the cell type being studied; and in the case of fibroblasts it could depend on their origin (e.g. resident fibroblasts vs fibrocyte-derived fibroblasts).

In summary, the current translational research on CSD subregions is novel because, to the best of our knowledge, this is the first study to demonstrate that the 82–89 and 94–101 subregions are active in vivo. Therefore, 82–89 and 94–101, like 88–95, may be the basis for novel treatments for the wide range of diseases involving fibrosis and/or microvascular leakage.

## Supporting information

S1 Fig IVRT. Tissue Doppler was used in the short-axis view at the papillary muscle level to calculate IVRT.
(TIF)

S2 Fig EF and FS. M-mode image of the parasternal short-axis view at the papillary muscle level used to calculate EF and FS.
(TIF)

S3 Fig SV and CO. B-mode Diastole/Systole images of the parasternal long-axis used to calculate SV and CO.
(TIF)

S1 Data (XLSX)

S2 Data (XLSX)

S3 Data (XLSX)

S1 Raw images (PDF)
