# Supplementary figures and images for: Multiple subregions within the caveolin-1 scaffolding domain inhibit fibrosis, microvascular leakage, and monocyte migration
Source: PLoS One. 2022 Feb 25;17(2):e0264413. doi: 10.1371/journal.pone.0264413 (PMC8880820; doi:10.1371/journal.pone.0264413)

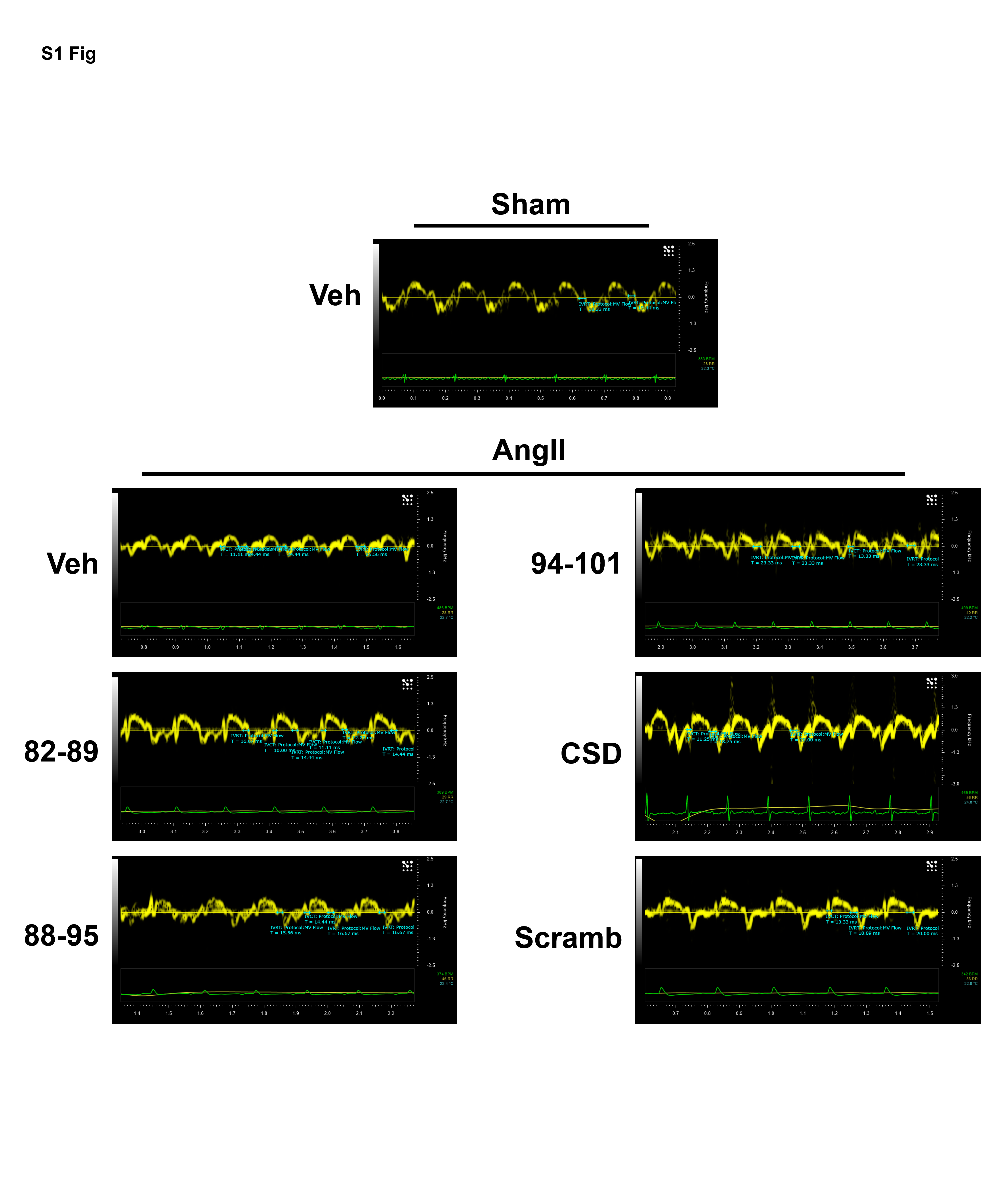

Supplement: S1 Fig — Tissue Doppler was used in the short-axis view at the papillary muscle level to calculate IVRT. (TIF) [file pone.0264413.s001.tif]

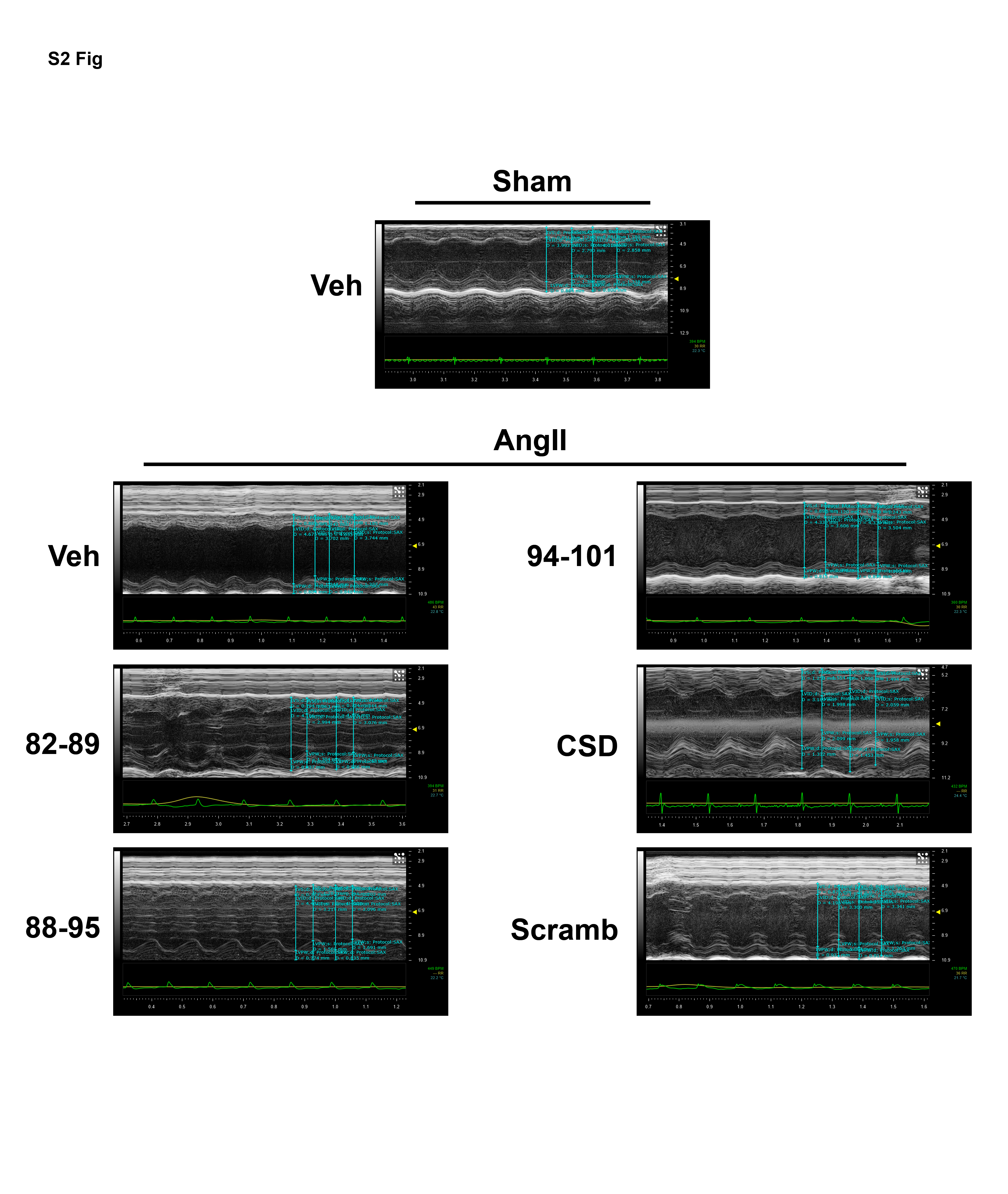

Supplement: S2 Fig — M-mode image of the parasternal short-axis view at the papillary muscle level used to calculate EF and FS. (TIF) [file pone.0264413.s002.tif]

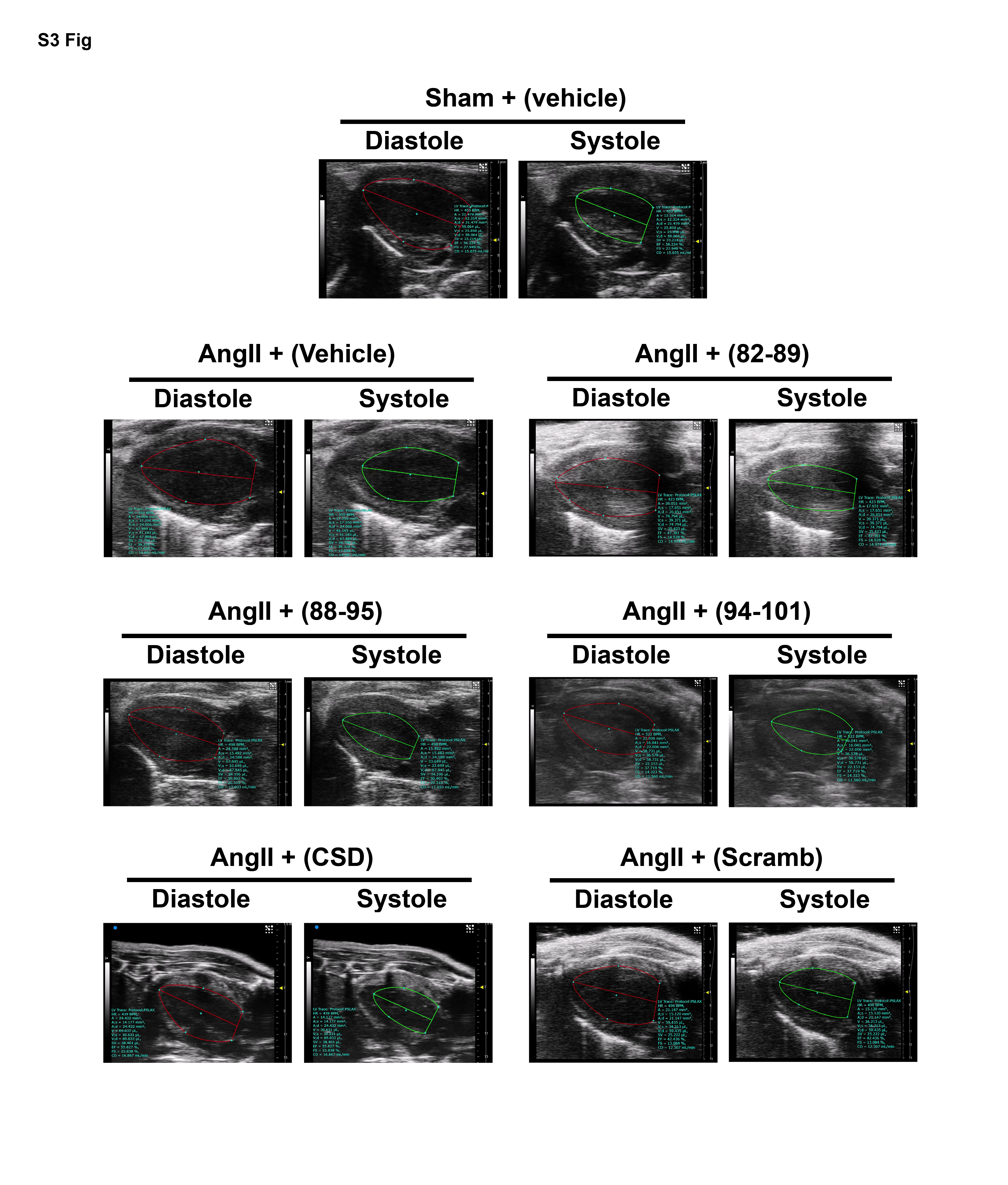

Supplement: S3 Fig — B-mode Diastole/Systole images of the parasternal long-axis used to calculate SV and CO. (TIF) [file pone.0264413.s003.tif]

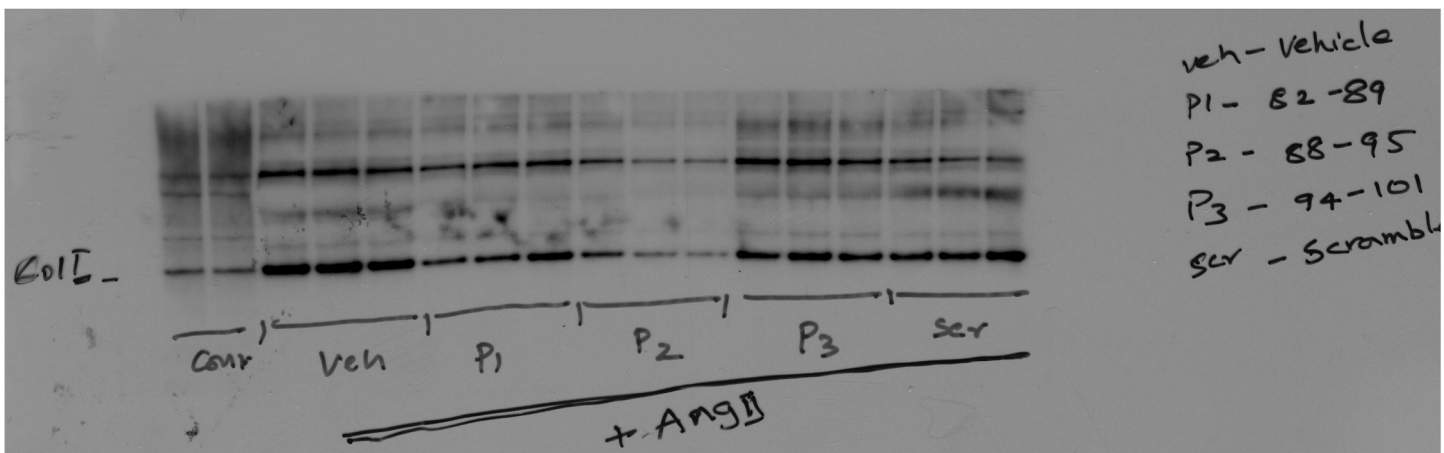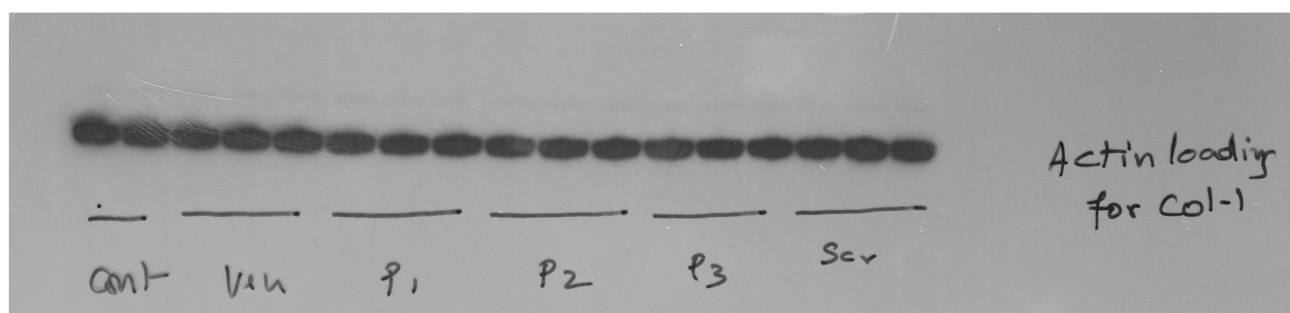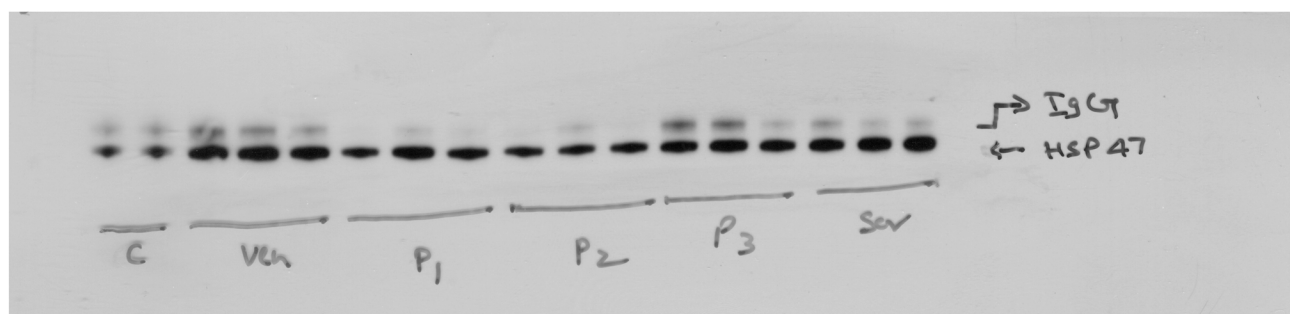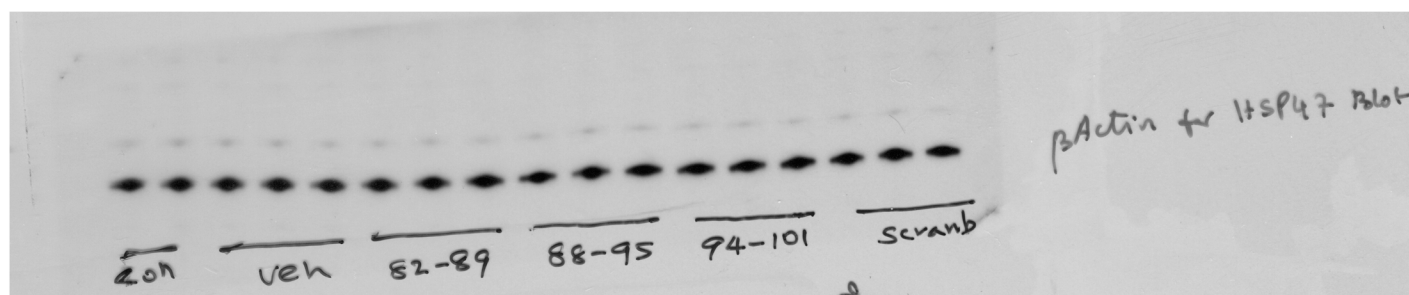

Supplement: S1 Raw images — (PDF) [file pone.0264413.s007.pdf]
